# Supplementary material for: Nifuratel reduces Salmonella survival in macrophages by extracellular and intracellular antibacterial activity
Source: Microbiol Spectr. 2023 Sep 21;11(5):e05147-22. doi: 10.1128/spectrum.05147-22 (PMC10581048; doi:10.1128/spectrum.05147-22)
Supplement: Supplementary Table 1 — Information of compound library with 66 drugs. [file spectrum.05147-22-s0001.docx]

**Supplementary Table 1.** Information of compound library with 66 drugs

| No. | Drug | Main function | No. | Drug | Main function |
| --- | --- | --- | --- | --- | --- |
| A2 | Cedryl acetate | α-glucosidase inhibitory activity | D5 | Magnolol | Antibacterial and antifungal agent |
| A3 | Hexetidine | Antibacterial and antifungal agent | D6 | Chloquinan | Antibacterial and antifungal agent |
| A4 | Berberine hydrochloride | Antibiotic | D7 | Spectinomycin dihvdrochloride | Antibiotic |
| A5 | Dichlorophen | Antimicrobial agent | D8 | Benzoic acid | Food preservative |
| A6 | Vagistat | Broad spectrum antifungal agent | D9 | Ethylparaben | Antimicrobial food preservative and antifungal agent |
| A7 | Amorolfine hydrochloride | Antifungal reagent | D10 | Thiabendazole | Antihelminthic |
| A8 | Sulbentine | Antifungal reagent | D11 | Trometamol | Biological buffer and alkalizer |
| A9 | Miconazole | Broad spectrum antifungal agent | E2 | Fluconazole | Broad spectrum antifungal agent |
| A10 | Crystal Violet | triarylmethane dye | E3 | Climbazole | Antifungal agent |
| A11 | Zinc pyrithione | Antifungal and antibacterial agent | E4 | Terbinafine hydrochlovide | Broad spectrum antifungal agent |
| B2 | Clotrimazole | Broad spectrum antifungal agent | E5 | Butoconazole nitrate | Antifungal agent |
| B3 | Miconazole nitrate | Antifungal agent | E6 | Ciclopirox | Broad spectrum antifungal agent |
| B4 | Fluorocytosine | Antifungal drug | E7 | Nifuratel | Antifungal agent |
| B5 | Amphotericin B | Antifungal agent | E8 | Nystatin | Antifungal antibiotic |
| B6 | Tolnaftate | Antifungal agent | E9 | Luliconazole | Broad spectrum antifungal agent |
| B7 | Ciclopirox ethanolamine | Antifungal agent | E10 | Pentamidine isethionate | antifungal and antiprotozoal agent |
| B8 | Imazalil | Topical antimycotic | E11 | Isavuconazole | Antifungal agent |
| B9 | Triclosan | Broad spectrum antifungal agent | F2 | Efinaconazole | Antifungal agent |
| B10 | Bifonazole | Topical antimycotic | F3 | Carbendazim | Broad spectrum antifungal agent |
| B11 | Butenafine hydrochloride | Antifungal agent | F4 | Myclobutanil | Fungicide |
| C2 | Sclareolide | Antifungal agent | F5 | Econazole nitrate | Antifungal agent |
| C3 | Gibberellic acid | Plant growth regulator | F6 | Pefloxacin mesylate dihydrate | Fungicide |
| C4 | Usnic acid | Antimicrobial and antitumor | F7 | Kasugamycin hydrochloride | Aminoglycoside antibiotic |
| C5 | Piroctone olamine | Antibacterial and antifungal agent | F8 | Voriconazole | Broad spectrum antifungal agent |
| C6 | Pravastatin sodium | Lipoprotein-lowering drug | F9 | Micafungin sodium | Antifungal drug |
| C7 | Pimaricin | Antifungal agent | F10 | Isoconazole nitrate | Broad spectrum antifungal agent |
| C8 | Salicylanilide | Antiviral, antibacterial and antifungal agent | F11 | Fenticonazole Nitrate | Broad spectrum antifungal agent |
| C9 | Zinc undecylenate | Antifungal agent | G2 | Sertaconazole nitrate | Broad spectrum antifungal agent |
| C10 | Clioquinol | Antifungal drug and antiprotozoal compound | G3 | Tavaborole | Antifungal agent |
| C11 | Chloroxylenol | Antimicrobial drug | G4 | Methyl 4-hydroxycinnamate | Anti-inflammatory agent and antifungal agent |
| D2 | Dehydroacetic acid | Antibacterial and antifungal agent | G5 | Terbinafine | Antifungal agent |
| D3 | Chlorobutanol | Preservative, sedative hypnotic and weak local anaesthetic | G6 | Sulconazole nitrate | Antifungal agent |
| D4 | Salicylic acid | Anti-inflammatory agent topical antibacterial agent | G7 | Tubercidin | Antiviral and antifungal agent |
